# Tribological Aspects of Cutting Tool Wear during the Turning of Stainless Steels

**DOI:** 10.3390/ma13010123

**Published:** 2019-12-26

**Authors:** Magdalena Zawada-Michałowska, Paweł Pieśko, Jerzy Józwik

**Affiliations:** Faculty of Mechanical Engineering, Lublin University of Technology, 20-618 Lublin, Poland

**Keywords:** tribology, cutting tool wear, turning, stainless steel

## Abstract

The research paper presents the tribological aspects of cutting tool wear during the turning of stainless steels. An experiment was conducted in order to assess the wear of carbide cutting inserts with CVD-applied anti-wear coatings (CNMG 12 04 08 ZSZ and CNMA 12 04 12-KR 3205) and an uncoated ceramic cutting insert (CNGA 12 04 08 T0102 WG 650). The test subject included the following stainless steel grades X20Cr13 (1.4021) and X8CrNiS18-9 (1.4305). The analysis involved the direct wear indicator, *VB_Bmax_*, and the indirect wear indicator, which was the roughness of machined surfaces and the *Ra* parameter. Based on the obtained results for both X20Cr13 and X8CrNiS18-9 steels, it was noticed that the best durability was exhibited by the CNMG 12 04 08 ZSZ insert, whereas the worst durability was identified for the CNGA 12 04 08 T0102 WG 650 insert. When analysing the results obtained with the *VB_Bmax_* direct indicator, it was observed that in the case of each of the insert, most often the course of their wear was of nature similar to linear. Comparing the *VB_Bmax_* direct indicator with the indirect indicator, namely, the measured *Ra* parameter, it was concluded that they were convergent. Upon the deterioration of the surface quality, greater values of the selected geometric measure of wear on the flank face were also identified.

## 1. Introduction

A cutting tool and a machined workpiece constitute a specific type of a tribological pair. The conditions prevailing within a cutting zone are characterized by high mechanical and thermal loads present at the contact point of the tool with the workpiece and chip, as well as high speeds between individual elements of the tribological pairs [1,2]. The main causes of wear are, therefore, thermal, mechanical and molecular actions between the edge, machined material, and chip. A cutting tool wear may be of mechanical (abrasive, strength-related, and plastic deformation), adhesive, chemical (diffusion, oxidation), and thermal nature [3,4,5,6,7,8,9]. It is most commonly associated with geometrical changes and varying physical properties of the tool under operation. The geometrical changes are caused by the friction that is related to loss of edge material, whereas the changing properties by local plastic deformation, high temperatures, and the chemical impact of the cooperating medium. When outside the recommended operating parameters, cases of catastrophic wear may appear, which result from locally exceeding the edge material strength [10,11,12]. In the case of machining stainless steels, the intensification of all types of wear is performed. Catastrophic wear is often arisen during machining such materials.

The tool wear is assessed using indicators, which should exhibit the following features [13]:

- Clearly illustrate the change of the geometry and properties of the tool material resulting from edge wear;
- Monotonically increase within the normal wear zone;
- Enable the execution of precise measurements.

In practice, the *VB* wear indicators for the flank face, the changes of which provide a view of the cutting capabilities of an edge, are adopted as indicators characterizing the wear. It should be noted that the wear of the cutting edge progressing over time, especially the wear appearing on the flank face, causes gradual deterioration of the machined surface quality, its roughness in particular [4,5,7,11,14,15,16,17,18,19,20]. Therefore, the roughness profile parameters are used as one of the so-called indirect wear indicators.

The type of wear during machining of stainless steels depends on material machinability. The stainless steels group includes materials with different machinability. Ferritic and martensitic steels are characterized by relatively good machinability, while high-alloy steels with austenitic structure are machined very poorly. The machinability of these steels is primarily influenced by the tendency toward strain hardening and low heat capacity and conductivity. For this reason, it is very important to choose the right cutting parameters and tool material as well as geometry [20].

The selection of edge geometry and the type of applied protective coating are particularly important in decreasing tool wear. Tool edge geometry should reduce the contact surface between the tool with the chip and the machined workpiece. It minimizes the friction occurring during cutting time, being one of the main factors impacting the tool edge durability, the reduction of the machined workpiece temperature, and the amount of heat transferred to the blade [16,21].

The use of tool coating fulfils several functions, the most important of which are [9,18,22,23,24]

- Increasing the hardness of the tool surface layer;
- Increasing the resistance to abrasive wear;
- Improving tribological properties (decreased adhesion);
- Increasing heat resistance.

The research was carried out in order to compare the effects of machining by a tool with an edge made of ceramic and tools with two types of carbide an edge. The starting point for the research was the assumption that the insert made of ceramic, due to its higher heat resistance, would be better than coated carbide inserts to use for the machining of stainless steels.

## 2. Materials and Methods

The aim of the research was to assess the tribological aspects of turning tool wear during the machining of selected stainless steel grades. A friction tribological pair was defined as an area of contact between the workpiece and the tool.

The turning was conducted with a DMG MORI CTX450 turning centre with a Sinumeric 840D control system. The samples were prepared from stainless steels widely used within the industry: X20Cr13 (1.4021) and X8CrNiS18-9 (1.4305). The materials were not heat treated. Table 1 presents chemical compositions of X20Cr13 and X8CrNiS18.9 stainless steels and Table 2—selected mechanical properties.

**Figure 1:**
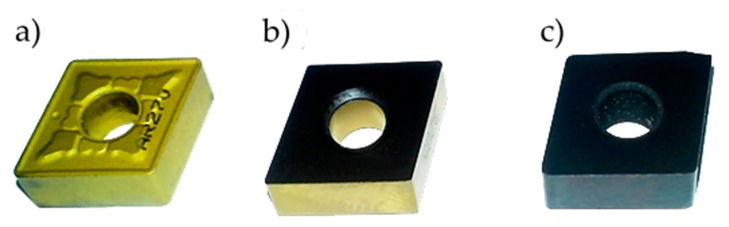
Indexable inserts used in the tests: (**a**) CNMG 12 04 08 ZSZ, (**b**) CNMA 12 04 12-KR 3205, and (**c**) CNGA 12 04 08 T0102 WG 650.

The following cutting inserts were used for the tests:

- Indexable insert (CNMG 12 04 08 ZSZ)—material: carbide with a CVD-applied TiN coating (Figure 1a)—material trade name: BP30A;
- Indexable insert (CNMA 12 04 12-KR 3205)—material: carbide with a CVD-applied Ti(C,N) + Al_2_O_3_ + TiN coating (Figure 1b)—material trade name: GC3205;
- Indexable insert (CNGA 12 04 08 T0102 WG 650)—material: mixed ceramic (Al_2_O_3_-based), without coating (Figure 1c)—material trade name: CC650.

The inserts used in the tests have a diamond shape (C shape) with a corner angle of 80° and a clearance angle of 0°.

A rolled bar with a diameter of 12 mm was the semi-finished product in the experiment. The cutting length was *l* = 150 mm. The succeeding cutting parameters were applied: depth of cut *a_p_* = 1 mm, cutting speed *v_c_* = 75 m/min, and feed *f* = 0.12 mm/rev. The tests were performed without cutting fluid (dry cutting), which is recommended due to its ecological aspects [27,28].

The research involved analysing the selected direct wear indicator, i.e., *VB_Bmax_*, and the indirect indicator, which was the roughness of machined surfaces, evaluated based on the *Ra* parameter (arithmetic mean of the roughness profile ordinates).

Roughness of the machined surface of rolled bars was measured with the Hommel Tester T1000 contact profilometer (Hommel, Villingen-Schwenningen, Germany). The *Ra* parameter was measured at three sample places, with five measurements for each of them, and the results were averaged. The insert cutting edge condition was assessed using a Keyence VHX-5000 digital microscope (Keyence, Itasca, MN, USA). Its capabilities also enabled the measuring of the edge wear. Insert wear was monitored after each tool pass (after each *l* = 150 mm). The tests were repeated five times for each configuration. The number of repetitions was determined from Equation (1) [29]:

(1) $$n=\frac{t_{\left(\alpha ;f\right)}^{2}S_{x}^{2}}{\varepsilon^{2}}$$

where $S_{x}^{2}$—variance of the tested variable determined on the basis of the preliminary test results, *ε*—accuracy of the assessment, *t*—critical value of the Student’s *t*-test, *α*—significance level, and *f*—number of degrees of freedom.

Observations of the wear behaviour and coating thickness of cutting inserts were performed using the Nova NanoSEM 450 scanning electron microscope of FEI (FEI, Hillsboro, OR, USA). A high vacuum and accelerating voltage of 15 kV were used. The measurements of coatings thickness were made on polished cross-sections of cutting inserts.

**Figure 2:**
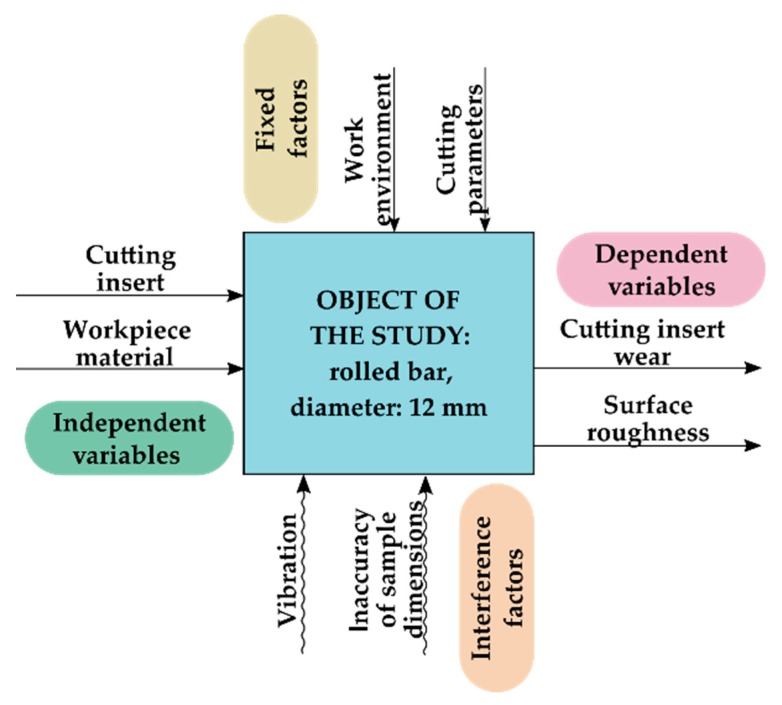
Research plan.

The research plan is shown in Figure 2. The independent variables were the cutting insert and the workpiece material, while the dependent variables were the cutting insert wear and the surface roughness. The fixed factors were the work environment and the cutting parameters, as well as the interferences were the vibration and the inaccuracy of sample dimensions.

Cutting was performed until edge damage was identified or worse surface quality was obtained.

## 3. Results

**Figure 3:**
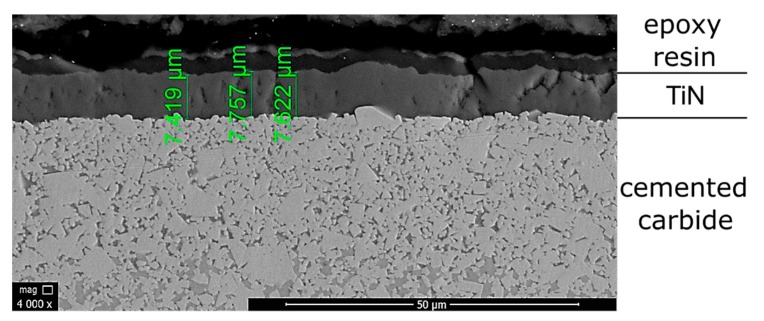
Coating thickness of CNMG 12 04 08 ZSZ carbide cutting insert (×4000).

**Figure 4:**
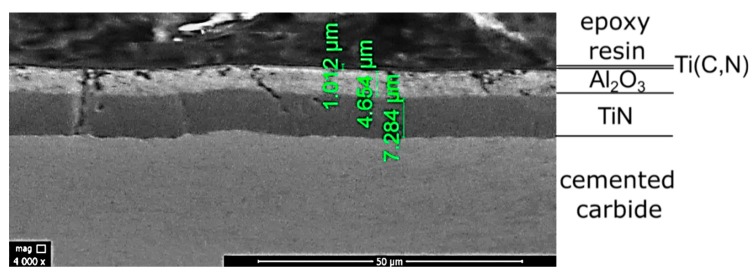
Coating thickness of CNMA 12 04 12-KR 3205 carbide cutting insert (×4000).

In the first instance, the thickness of coatings used in carbide cutting inserts was determined. Figure 3 and Figure 4 present SEM micrographs of coatings thickness of tested CVD-coated carbide cutting inserts. The estimated coating thickness of CNMG 12 04 08 ZSZ insert is about 7 μm. For CNMA 12 04 12-KR 3205, the sum of three layers equals approximately 13 μm.

**Figure 5:**
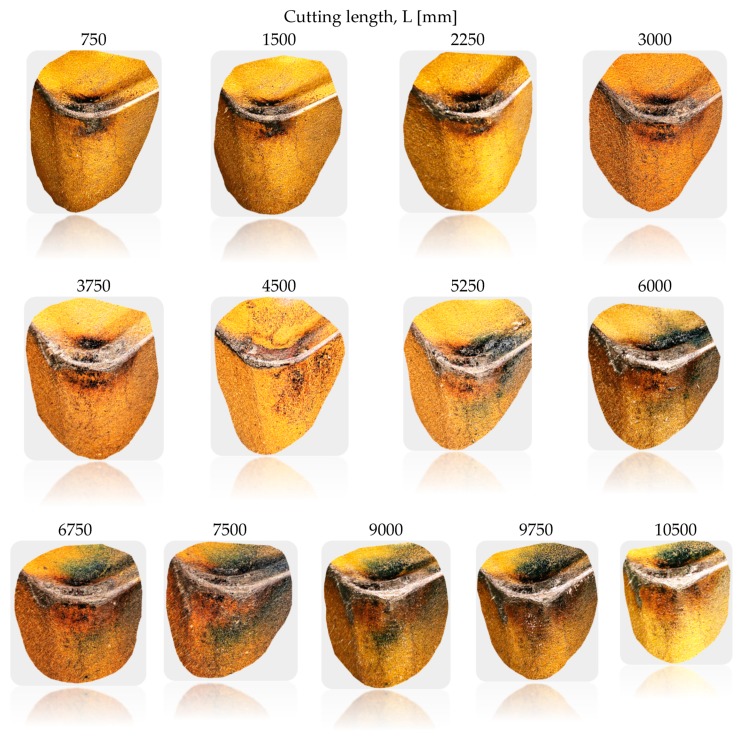
Exemplary wear phases of CNMG 12 04 08 ZSZ indexable insert for X8CrNiS18-9 stainless steel depending on the cutting length *L* [20].

The research involved analysing the impact of the cutting edge wear on the changes of the *Ra* parameter and selected direct wear indicator, *VB_Bmax_*, for three cutting inserts and two stainless steel grades. Figure 5 presents exemplary wear phases of CNMG 12 04 08 ZSZ indexable insert for X8CrNiS18-9 stainless steel depending on the cutting length *L*.

**Figure 6:**
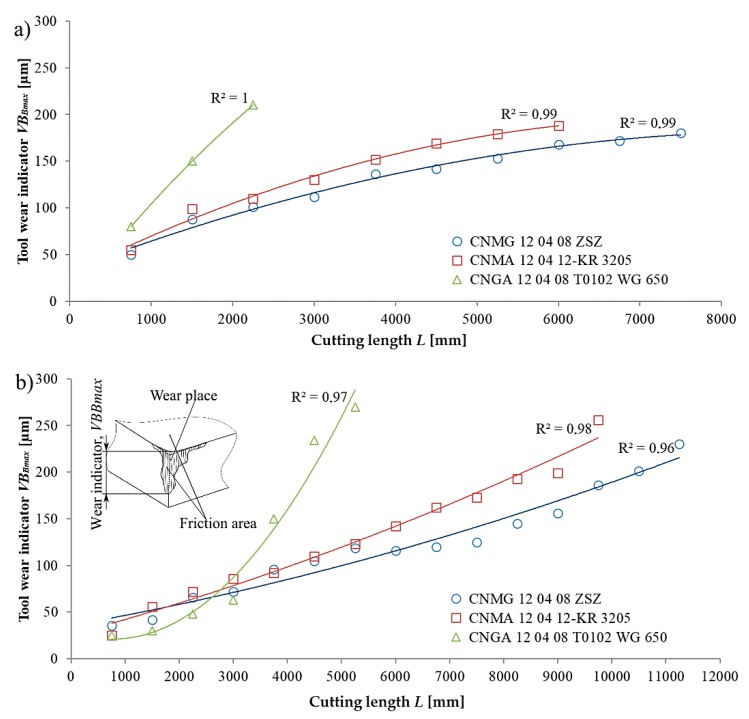
Course of wear for the *VB_Bmax_* indicator as a function of the cutting length *L* obtained for the following cutting inserts: CNMG 12 04 08 ZSZ, CNMA 12 04 12-KR 3205, and CNGA 12 04 08 T0102 WG 650, and the studied stainless steels (**a**) X20Cr13 and (**b**) X8CrNiS18-9.

The *VB_Bmax_* indicator was determined as a cutting-length function *L*. Different cutting lengths *L* for individual inserts result from their varying durability. Figure 6 shows the measurement results for the *VB_Bmax_* indicator obtained for two stainless steels, X20Cr13 and X8CrNiS18-9, and the tested cutting inserts.

The test results for *VB_Bmax_* indicator were approximated with a second-degree polynomial function. Equations (2) and (5) show the trend of *VB_Bmax_* for the CNMG 12 04 08 ZSZ insert. Equations (3) and (6) present the trend of *VB_Bmax_* for the CNMA 12 04 12-KR 3205 insert. Equations (4) and (7) show the trend of *VB_Bmax_* for the CNGA 12 04 08 T0102 WG 650 insert.

X20Cr13 stainless steel

(2) $$VB_{Bmax}=9\times 10^{-8}L^{2}k_{2}-0.0005Lk_{1}+1.9906k_{0}$$

(3) $$VB_{Bmax}=10^{-7}L^{2}k_{2}-0.0006Lk_{1}+1.7203k_{0}$$

(4) $$VB_{Bmax}=10^{-6}L^{2}k_{2}-0.0057Lk_{1}+1.965k_{0}$$

X8CrNiS18-9 stainless steel

(5) $$VB_{Bmax}=-2\times 10^{-9}L^{2}k_{2}+9\times 10^{-5}Lk_{1}+1.3206k_{0}\;$$

(6) $$VB_{Bmax}=4\times 10^{-9}L^{2}k_{2}+0.0001Lk_{1}+1.3583k_{0}$$

(7) $$VB_{Bmax}=3\times 10^{-7}L^{2}k_{2}-0.001Lk_{1}+2.553k_{0}$$

where *k_i_*, representing the polynomial unit factors, corresponds to the unit ordinals $k_{2}=1\frac{\mu m}{{\mathrm{mm}}^{2}},k_{1}=1\frac{\mu m}{\mathrm{mm}},\;\mathrm{and}\;k_{0}=1\;\mu m$.

Based on the presented results pertaining to the *VB_Bmax_* direct indicator, it was concluded that in the case of each of the inserts, the course of the wear was of a linear-like nature. For the CNGA 12 04 08 T0102 WG 650 insert, which due to its material is susceptible to vibrations of the machine tool-workpiece-fixture-tool system, it was found to have approximately 60% and 45% shorter cutting length than for the CNMA 12 04 12-KR 3205 insert during turning X20Cr13 and X8CrNiS18, respectively. It was the result of quickly appearing catastrophic wear in the form of cutting edge crushing in the case of CNGA 12 04 08 T0102 WG 650 insert. An analogical reliance was noted in relation to CNMG 12 04 08 ZSZ; however, differences amounted to 70% and 55%. Comparing CNMA 12 04 12-KR 3205 and CNMG 12 04 08 ZSZ, which had higher durability of 25% and 15% in reference to X20Cr13 and X8CrNiS18-9, produced CNMG 12 04 08 ZSZ.

**Figure 7:**
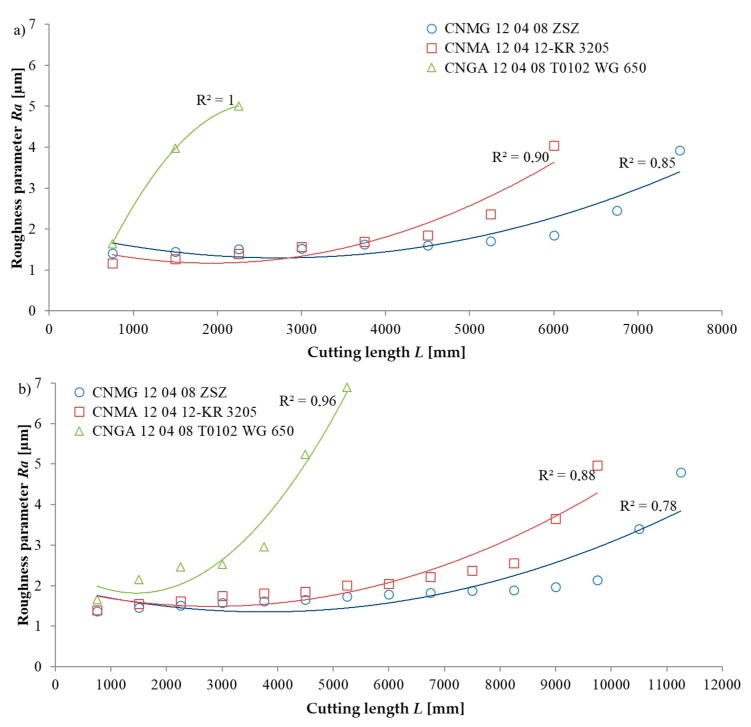
Changes of the Ra roughness parameter value obtained when turning with the following cutting inserts: CNMG 12 04 08 ZSZ, CNMA 12 04 12-KR 3205, CNGA 12 04 08 T0102 WG 650, and the studied stainless steels (**a**) X20Cr13 and (**b**) X8CrNiS18-9.

Figure 7 shows the values of the *Ra* roughness parameter obtained during turning X20Cr13 and X8CrNiS18 stainless steel grades with three cutting inserts. The *Ra* surface roughness parameter was also shown as a function of the cutting length *L*. When monitoring the condition of the cutting edge, its significant wear, preventing further machining, in each case translated to a sudden increase of the *Ra* roughness parameter value.

The test results for *Ra* indicator were also approximated with a second-degree polynomial function. Equations (8) and (11) present the trend of *Ra* for the CNMG 12 04 08 ZSZ insert. Equations (9) and (12) show the trend of *Ra* for the CNMA 12 04 12-KR 3205 insert. Equations (10) and (13) present the trend of *Ra* for the CNGA 12 04 08 T0102 WG 650 insert.

X20Cr13 stainless steel:

(8) $$Ra=-9\times 10^{-6}L^{2}k_{2}+0.1133Lk_{1}-6\times 10^{-13}k_{0}$$

(9) $$Ra=-3\times 10^{-6}L^{2}k_{2}+0.0434Lk_{1}+29.357k_{0}$$

(10) $$Ra=-2\times 10^{-6}L^{2}k_{2}+0.0333Lk_{1}+33.133k_{0}$$

X8CrNiS18-9 stainless steel:

(11) $$Ra=10^{-5}L^{2}k_{2}-0.0206Lk_{1}+29.143k_{0}$$

(12) $$Ra=6\times 10^{-7}L^{2}k_{2}+0.0157Lk_{1}+25.804k_{0}\;$$

(13) $$Ra=5\times 10^{-7}L^{2}k_{2}+0.0103Lk_{1}+35.774k_{0}$$

where *k_i_*, representing the polynomial unit factors, corresponds to the unit ordinals $k_{2}=1\frac{\mu m}{{\mathrm{mm}}^{2}},\;k_{1}=1\frac{\mu m}{\mathrm{mm}},\;k_{0}=1\;\mu m$.

Based on the obtained results for both the X20Cr13, as well as the X8CrNiS18-9 steels, a step-wise increase of surface roughness upon the appearance of significant cutting edge wear was noted. Comparing the values of the *Ra* parameter for individual inserts, it was concluded that the worst quality was characteristic for the surface after turning with the CNGA 12 04 08 T0102 WG 650 insert and the best quality was characteristic after the CNMG 12 04 08 ZSZ insert. Verifying the direct and indirect indicators, it can be concluded that they are convergent. Upon the deterioration of the surface quality, also greater values of the selected geometric measure of wear on the flank face were identified.

**Figure 8:**
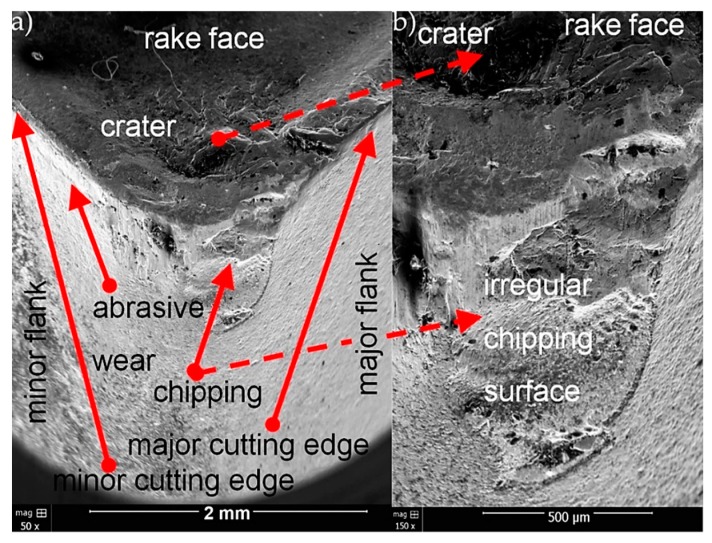
SEM micrographs of wear for CNMG 12 04 08 ZSZ insert with TiN coating for two magnifications (**a**) 50× and (**b**) 150×.

**Figure 9:**
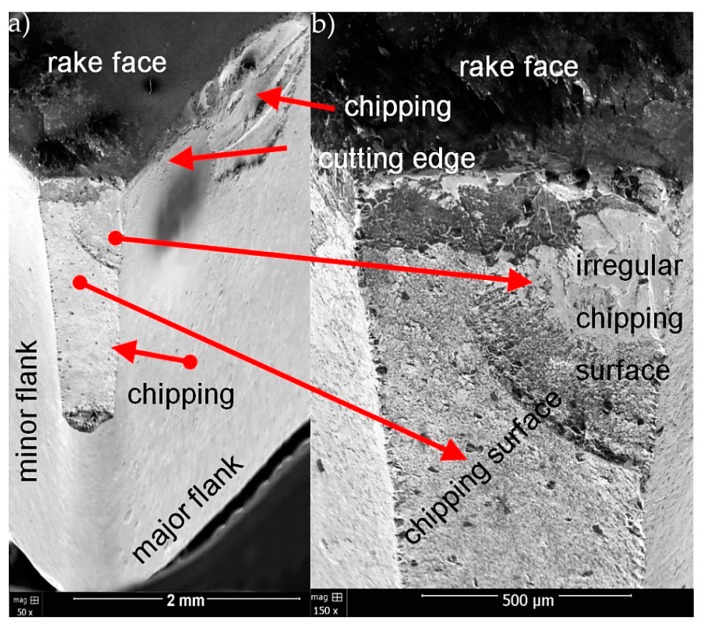
SEM micrographs of wear for CNMA 12 04 12-KR 3205 insert with Ti(C,N) + Al_2_O_3_ + TiN coating for two magnifications (**a**) 50× and (**b**) 150×.

**Figure 10:**
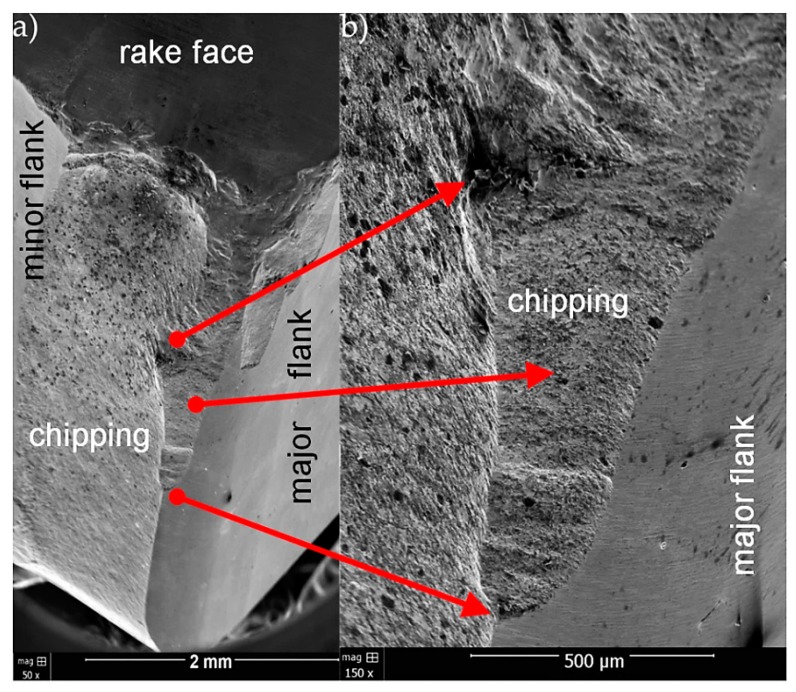
SEM micrographs of wear for CNGA 12 04 08 T0102 WG 650 insert without coating for two magnifications (**a**) 50× and (**b**) 150×.

Figure 8, Figure 9 and Figure 10 present SEM micrographs of wear for tested cutting inserts.

The abrasive character was the dominant of wear mechanism for CNMG 12 04 08 ZSZ insert with TiN coating (Figure 8). Progressive abrasive wear led to the destruction of TiN coating and corner weakness. As a consequence, the corner was broken in the final phase. The chipping surface has an irregular surface structure. Crater on the rake face was also observed.

For CNMA 12 04 12-KR 3205 insert with Ti(C,N) + Al_2_O_3_ + TiN coating, apart from chipping of the corner (similar to CNMG 12 04 08 ZSZ insert), the cutting edge was wiped and chipped (Figure 9).

In the case of CNGA 12 04 08 T0102 WG 650 insert, as in the previous cases, in the final stage of machining, the cutting wedge was catastrophically dulled and, as a consequence, the corner was broken along the entire thickness of the insert (Figure 10).

Analysing cutting edge wear for CNMG 12 04 08 ZSZ insert, it was found that abrasive wear and, eventually, chipping appeared, and for CNMA 12 04 12-KR 3205 and CNGA 12 04 08 T0102 WG 650 inserts, chipping wear appeared. The differences in the obtained values of the *VB_Bmax_* wear indicator and the surface roughness *Ra* result from the dissimilar wear mechanism of the CNMG 12 04 08 ZSZ compared to the other two inserts.

The inserts also differ in geometry. The CNMG 12 04 08 ZSZ has hip breakers, while the other inserts have a flat rake face, which results in different values and distributions of cutting force components.

## 4. Conclusions

The obtained test results allowed one to formulate the following conclusions:

- For the CNGA 12 04 08 T0102 WG 650 insert, during machining of X20Cr13 and X8CrNiS18, respectively, about 60% and 45% shorter cutting length than for the CNMA 12 04 12-KR 3205 was noted;
- It was also found that with reference to CNMG 12 04 08 ZSZ insert, for CNGA 12 04 08 T0102 WG 650 about 70% and 55% shorter cutting length was received for both stainless steels;
- Comparing CNMA 12 04 12-KR 3205 and CNMG 12 04 08 ZSZ, a higher durability of 25% and 15% in reference to X20Cr13 and X8CrNiS18-9 showed CNMG 12 04 08 ZSZ;
- Verifying the direct and indirect indicators, it can be concluded that they are comparable. It is related to visible deterioration of surface quality at the moment of increasing the value of the selected wear indicator *VB_Bmax_*;
- During machining of both the X20Cr13, as well as the X8CrNiS18-9 steels, a step-wise increase of surface roughness upon the appearance of significant cutting edge wear was noted;
- The worst surface quality was obtained after turning with the CNGA 12 04 08 T0102 WG 650 insert, while the best surface quality was obtained after the CNMG 12 04 08 ZSZ insert;
- The differences in the obtained values of the *VB_Bmax_* wear indicator and the surface roughness *Ra* result from the dissimilar wear mechanisms of CNMG 12 04 08 ZSZ compared to the other two inserts;
- Cutting edge wear for CNMG 12 04 08 ZSZ insert was abrasive, and chipping eventually appeared. However, for the CNMA 12 04 12-KR 3205 and CNGA 12 04 08 T0102 WG 650 inserts, the wear had a chipping character;
- The inserts also differ in geometry. The CNMG 12 04 08 ZSZ insert has chip breakers, while the other inserts have a flat rake face, which results in different values and distributions of cutting force components.

Considering the durability of replaceable indexable cutting inserts, a CNMG 12 04 08 ZSZ insert is best suited for the machining of the tested stainless steels.

## Figures and Tables

**Table 1:** Chemical compositions of X20Cr13 and X8CrNiS18.9 steels [25,26].

Chemical Composition [%]								
**X20Cr13 (1.4021)**								
**C**	**Cr**	**Ni**	**Si**	**Mn**	**P**	**S**	**N**	**Cu**
0.16–0.25	12–14	-	<1	<1.5	<0.04	<0.015	-	-
**X8CrNiS18-9 (1.4305)**								
<0.1	17–19	8–10	<1	<2	<0.045	0.15–0.35	<0.11	<1

**Table 2:** Mechanical properties of X20Cr13 and X8CrNiS18.9 steels [25,26].

Mechanical Properties			
Indication	Yield Strength, *R_p_*_0.2_ [MPa]	Tensile Strength, *R_m_* [MPa]	Brinell Hardness, [HB]
X20Cr13 (1.4021)	345	<700	225
X8CrNiS18-9 (1.4305)	190	520–700	190
